# Lung microvasculopathy in chronic thromboembolic pulmonary hypertension: high-resolution findings with photon-counting detector CT in 29 patients

**DOI:** 10.1007/s00330-025-11561-w

**Published:** 2025-04-18

**Authors:** Martine Remy-Jardin, Alain Duhamel, Marie Delobelle, Jean-François Bervar, Thomas Flohr, Jacques Remy

**Affiliations:** 1 Department of Thoracic Imaging, University of Lille, Lille, France; 2 ULR 2694 METRICS Evaluation des Technologies de santé et des Pratiques Médicales, LILLE, France; 3 IMALLIANCE-Haut-de-France, Valenciennes, France; 4 Department of Biostatistics, University of Lille, CHU Lille, Lille, France; 5 Department of Cardiology, University of Lille, Lille, France; 6 Department of Pulmonology, University of Lille, Lille, France; 7 Department of Computed Tomography Research & Development, Siemens Healthineers AG, Forchheim, Germany; 8 Department of Radiology and Nuclear Medicine, Maastricht University Medical Centre, Maastricht, The Netherlands; 9 Department of Radiology, Valenciennes Regional Hospital, Valenciennes, France

**Keywords:** Chronic thromboembolic disease, Pulmonary hypertension, Pulmonary arteries, Computed tomography, Photon-counting-detector CT

## Abstract

**Purpose:**

To evaluate CT findings suggestive of lung microvasculopathy in patients with chronic thromboembolic pulmonary hypertension (CTEPH).

**Materials and methods:**

Twenty-nine patients were scanned with high-spatial resolution on a photon-counting detector (PCD)-CT unit. A maximum of three pairs per patient, each composed of hyper- and hypo-attenuating areas of mosaic perfusion, were selected.

**Results:**

Comparative analysis of the 86 selected pairs showed: (a) a higher frequency of ill-defined micronodules (*p* = 0.008), lobular ground-glass opacities (*p* = 0.01) and haziness (*p* = 0.003) in hypoattenuated areas; (b) there was no significant difference in the frequency of neovascularity (*p* = 0.43). Similar trends were observed in hypoattenuating areas of the 66 pairs studied in the 22 patients with central and peripheral CTEPH; an absence of ill-defined micronodules, lobular ground-glass opacities, and haziness in hyperattenuating areas was noticed in the 20 pairs studied in the 7 patients with peripheral CTEPH. Patients with a mean pulmonary artery pressure ≤ 42 mmHg (i.e., the median value of mean pulmonary artery pressure) had 45 pairs compared, showing a higher frequency of ill-defined micronodules (*p* = 0.003) and haziness (*p* < 0.001) in hypoattenuated areas, together with a higher frequency of subpleural systemic-to-pulmonary anastomoses (*p* = 0.02). There were no statistical differences in the frequency of CT findings between hypo- and hyper-attenuating areas in the 41 pairs of patients with a mean pulmonary artery pressure > 42 mm Hg.

**Conclusion:**

CT features suggestive of microvasculopathy were more frequent in areas of hypoperfusion, with a trend toward homogenization of CT findings in patients with severe PH.

**Key Points:**

***Question*** *Lung microvascular lesions play a crucial role in the origin of residual pulmonary hypertension after successful thromboendarterectomy, currently beyond the scope of imaging*.

***Findings*** *The expected morphological abnormalities at the level of distal pulmonary circulation in CTEPH were found to be depictable in each zone of mosaic perfusion*.

***Clinical relevance*** *This study suggests that the high-spatial resolution of PCD-CT has the capability of approaching the complex pathophysiology of small-vessel disease in CTEPH, providing important information prior to therapeutic decisions*.

**Graphical Abstract:**

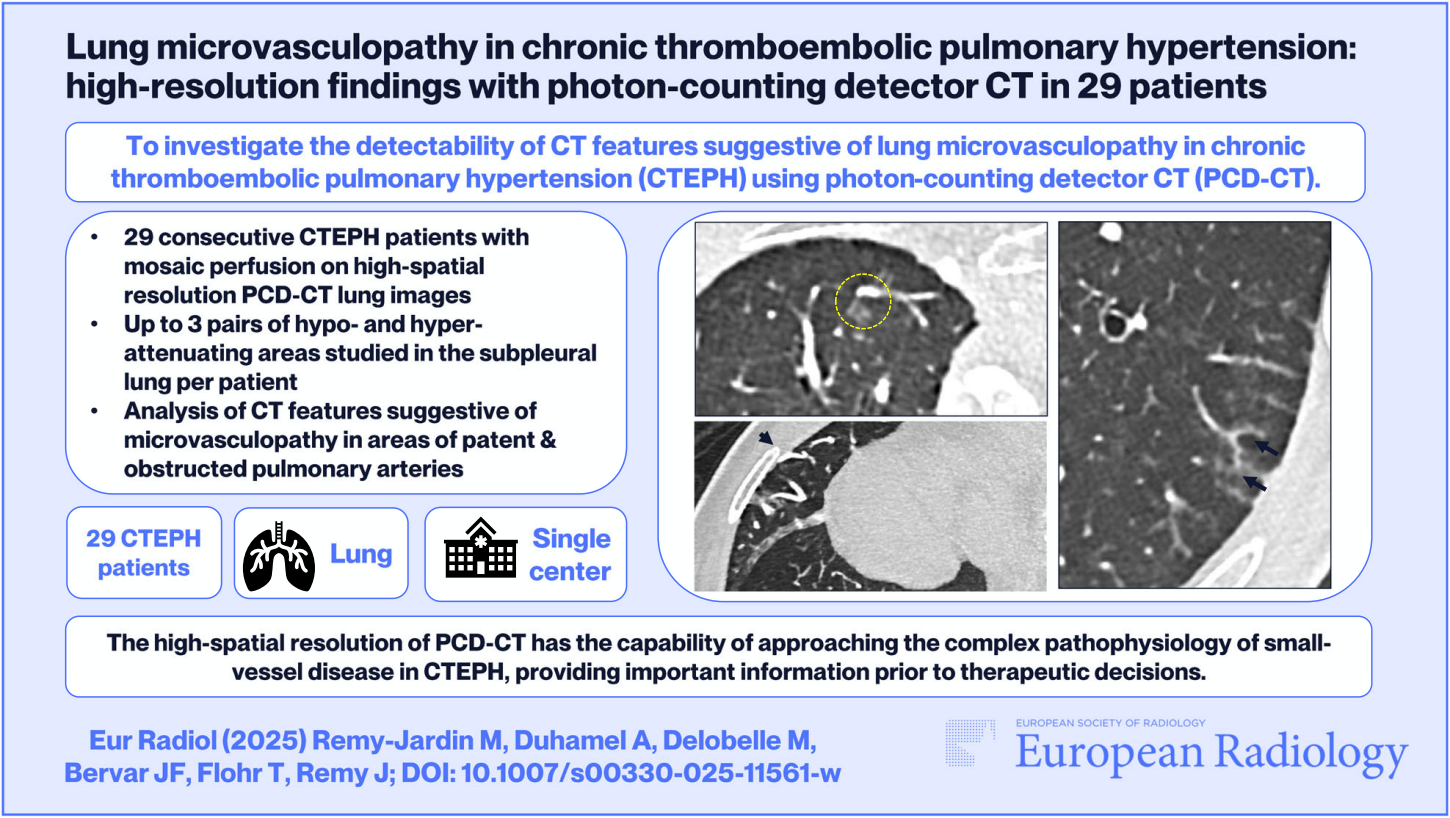

## Introduction

Chronic thromboembolic pulmonary hypertension (CTEPH) is a progressive disease, resulting from the fibrotic transformation of unresolved clots within central and peripheral pulmonary arteries, combined with variable degrees of small-vessel arteriopathy. Whereas central and peripheral lesions are targets of pulmonary thromboendarterectomy (PEA) and/or balloon pulmonary angioplasty (BPA), small-vessel arteriopathy is inaccessible to surgery or interventional procedures. Involving pulmonary arterioles, venules, and capillaries, the small-vessel arteriopathy includes vascular remodeling secondary to the redistribution of blood flow in nonoccluded pulmonary arteries, whereas more complex lesions are observed distally to chronically obstructed pulmonary arteries, secondary to the development of a systemic blood supply to ischemic lung regions [1]. These microvascular lesions contribute to elevated pulmonary vascular resistance and are considered to play a crucial role in the origin of residual pulmonary hypertension (PH) after successful PEA [2–4]. They also support the use of pulmonary artery hypertension (PAH) medications to treat CTEPH microvascular disease [5].

Whereas chronic obstruction of central and peripheral pulmonary arteries is accessible to morphology and functional imaging [6], small-vessel arteriopathy is currently considered beyond the scope of imaging. Several indirect clues suggest the presence of extensive small-vessel disease, suspected when the extent of proximal fibrotic clots does not correlate with the hemodynamic severity or in the presence of disproportionally poor hemodynamics with limited scintigraphic perfusion defects [7–9]. Over the last decade, a special aspect of diffuse, poor subpleural perfusion (PSP) has raised great interest in suggesting small-vessel disease in CTEPH. PSP in the capillary phase of digital pulmonary angiograms was found to be a predictor of poor outcome of pulmonary endarterectomy for operable CTEPH and BPA for non-operable CTEPH [10, 11]. On CT lung perfusion maps, patients exhibiting this finding had higher pulmonary vascular resistance (PVR) and lower diffusing capacity of the lungs for carbon monoxide (DLCO) than patients with normally perfused subpleural space [12]. A quantitative approach to lung perfusion in the subpleural lung has recently been investigated in different subtypes of PH, CTEPH, showing a reduced volume of small pulmonary vessels [13].

However, there has been no attempt to provide morphologic analysis of small pulmonary vessels, which is considered to fall below the limits of resolution of clinical CT scanning. The introduction of high-spatial resolution scanning with photon-counting-detector CT (PCD-CT) opens new areas of investigation, currently mainly used to improve analysis of subtle features of lung fibrosis [14–16]. In the context of CTEPH, this raises an obvious interest in the potential detectability of subtle changes in the most distal part of the pulmonary circulation in occluded and non-occluded areas, visually recognized when a mosaic perfusion pattern can be detected on CT lung images. This pattern is defined by the presence of areas of ground-glass attenuation with enlarged vascular sections (i.e., areas of redistribution of blood flow), intermingled with areas of low attenuation with smaller vascular sections (i.e., areas of obstructed pulmonary arteries) [17, 18], highlighting the hypo- and hyperperfused lung areas in CTEPH [19, 20].

The main objective of this study was to investigate whether the expected morphological findings in lung microcirculation of CTEPH patients could be detected on PCD-CT lung images. The secondary objective was to search for potential differences in CT findings between areas of hypo- and hyper-attenuation of the mosaic perfusion pattern.

## Materials and methods

### Population

**Fig. 1:**
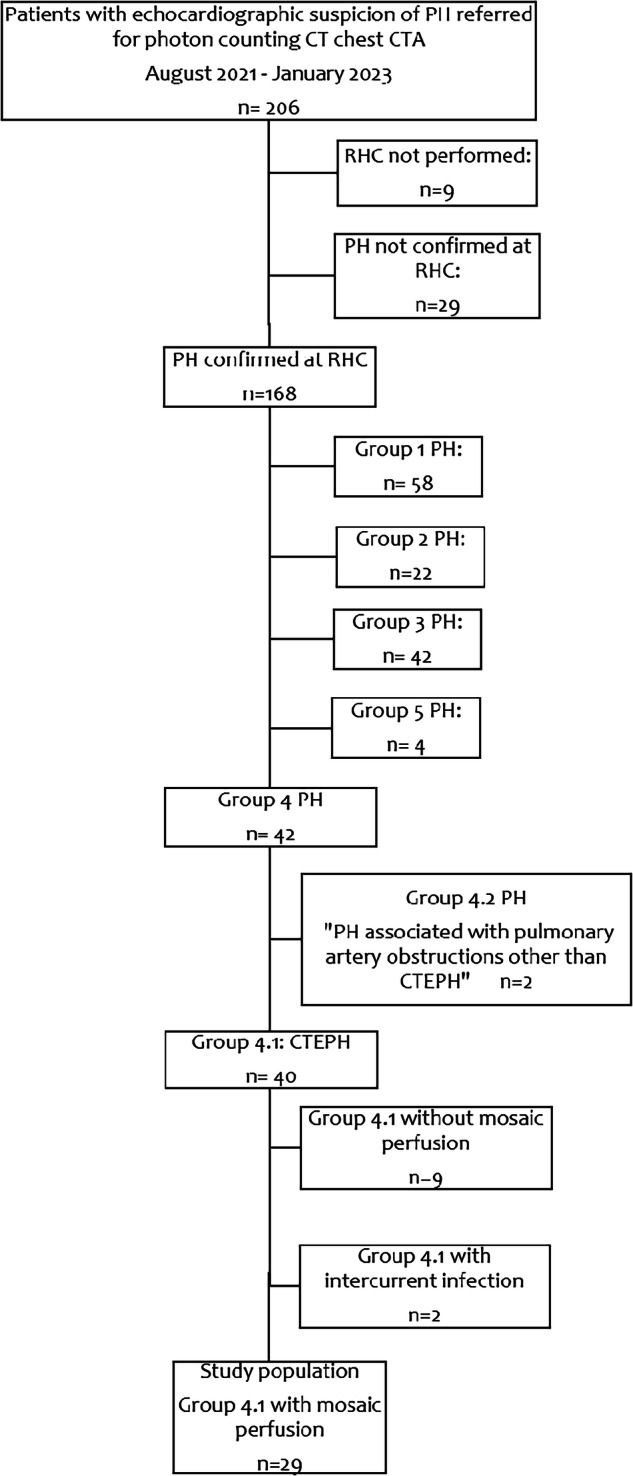
Flow-chart of the study population. PH, pulmonary hypertension; CTA, computed tomography angiography; RHC, right heart catheterization; CTEPH, chronic thromboembolic pulmonary hypertension; Group 1 PH, pulmonary arterial hypertension; Group 2 PH, PH associated with left heart disease; Group 3 PH, PH associated with lung diseases and/or hypoxia; Group 4 PH, PH associated with pulmonary artery obstructions; Group 5 PH, PH with unclear and/or multifactorial mechanisms

This retrospective, non-interventional study was approved by the institutional review board with waiver of patients’ informed consent in accordance with national regulations after validation by the local Ethics Committee. The study population was selected from a cohort of 206 consecutive patients with echocardiographic suspicion of PH who had been referred for chest CTA during a period extending from between August 2021 (i.e., date of installation of the PCD-CT equipment in the radiology department) and January 2023 (i.e., end of the inclusion for the present investigation). As summarized in Fig. 1, the study population fulfilled the following criteria: (a) PH confirmed by right-heart catheterization; (b) PH etiology exclusively limited to CTEPH, thus excluding patients diagnosed with Groups 1, 2, 3, and 5 PH and Group 4 patients with non-CTEPH pulmonary artery obstruction; CTEPH diagnosis was assessed during multidisciplinary meetings including PH experts (cardiologists, pulmonologists; radiologists); and (c) CTEPH patients without intercurrent disease in whom chest CTA showed a mosaic perfusion pattern. The final study population consisted of 29 patients whose clinical and hemodynamic characteristics are summarized in Table 1.

**Table 1:** Clinical and hemodynamic characteristics of the study population

Females/males, *n* (%)	15 (51.7%)/14 (48.3%)
Age, years mean ± SD	68.7 ± 12.7
NYHA functional classes	
I, *n* (%)	–
II, *n* (%)	15 (50.0%)
III, *n* (%)	13 (46.4%)
IV, *n* (%)	1 (3.6%)
6 MWT, mean ± SD	298.2 ± 134.0
DLCO, % mean ± SD	67.0 ± 16.9
Right heart catheterization	
Mean PAP, mm Hg	44.4 ± 10.1
Systolic PAP, mm Hg	75.4 ± 19.5
Diastolic PAP, mm Hg	27.4 ± 6.8
PAWP, mm Hg	8.3 ± 3.5
PVR, Wood unit	7.9 ± 3.2
Cardiac output, L/min	4.9 ± 1.2
Cardiac index, L/min/m^2^	2.6 ± 0.6
SVO2, %	61.7 ± 7.0
PH medical treatment	
No treatment, *n* (%)	22 (75.9%)
Monotherapy, *n* (%)	3 (10.3%)
Double combination, *n* (%)	3 (10.3%)
Triple combination, *n* (%)	1 (3.4%)

NB: categorical variables were expressed as frequencies and percentages, and quantitative variables were expressed as mean ± standard deviation

*NYHA* New York Heart Association, *6MWT* six-minute walk test, *DLCO* diffusing capacity of the lungs for carbon monoxide, *PAP* pulmonary artery pressure, *PAWP* pulmonary artery wedge pressure, *PVR* pulmonary vascular resistance, *SVO2* mixed venous oxygen saturation, *PH* pulmonary hypertension

### CT evaluation

To provide lung images with the highest spatial resolution, the scanning protocol was adapted to the technological evolution of our CT unit since its implementation as an early commercial product in August 2021. Twenty CTEPH patients were scanned with the thinnest collimation (i.e., 120 × 0.2 mm) that was: (a) initially only available for non-contrast, ultra-high-resolution (UHR) acquisitions (*n* = 9) completed by standard single-source CT angiograms; (b) included in UHR CT angiographic protocols when UHR spectral data acquisitions became available (*n* = 11). For dyspneic patients (*n* = 9), lung images were generated from acquisitions ensuring the shortest scanning time; the selected collimation was then 144 × 0.4 mm. A detailed description of the scanning parameters is summarized in Table A (Supplementary Material).

Lung images were reconstructed (a) as polychromatic images (0.2 mm) or at 70-keV (0.4 mm) with the same sharp lung kernel (Bl60); (b) the level of iterative reconstruction was QIR4 (Quantum Iterative Reconstruction) (range of QIR level: 1–4) for non-contrast images and QIR 3 for contrast-enhanced images. Two additional sets of images were systematically reconstructed: (a) mediastinal images, to provide a morphologic description of CTEPH-related vascular changes (central/peripheral, uni/bilateral pulmonary artery obstruction; abnormal dilatation of bronchial and non-bronchial systemic arteries); (b) perfusion images from spectral data acquisitions, to help confidently differentiate areas of hypo- and hyper-perfusion in case of mild gradient of attenuation on lung images, using a previously described methodology [21]. The mean (±SD) dose-length-product of PCD-CT examinations was 271.5 ± 19.09 mGy·cm.

### Analysis of distal lung circulation

#### Selection of target areas on lung images

A target area consisted of two adjacent zones (i.e., an area of hypo- and hyper-attenuation) in the same lobe, further referred to as a “pair”.

The criteria for selecting a pair included the following: (a) absence of motion artifacts (cardiogenic/respiratory/nearby pleural effusion); (b) clear lung, in particular absence of sequelae of pulmonary infarction or features of chronic lung ischemia. The two zones of a pair (a) were depictable on the same transverse CT section; (b) were seen as adjacent areas; (c) had no predefined surface (itself depending on the extent of chronic arterial obstruction) but included several adjacent secondary pulmonary lobules to allow analysis of the most distal anatomical structures depictable on CT sections; and (d) were selected in the subpleural lung of a given lobe.

The total number of pairs selected per patient was obtained as follows: (a) whenever possible, three pairs in the right lung; (b) when a right-sided selection was incomplete, the missing pair was selected in the left lung. Because of the uniform pathophysiology of CTEPH microvasculopathy and the exploratory nature of this study, we arbitrarily chose to select one pair per lobe.

#### CT features analyzed in each pair

In the absence of preliminary investigation, we extrapolated the list of CT features potentially detectable in obstructed and non-obstructed areas from pathophysiological descriptions as detailed in Table B (Supplementary Material). In the subpleural lung of each pair (i.e., 2–3 cm from the lung surface) analyzed on transverse CT sections: (a) we visually assessed the diameter of pulmonary arterioles (rated as thin or dilated) and venules (rated as thin, dilated or unremarkable), and the presence of septal lines (i.e., boundaries of secondary pulmonary lobules) when depictable; there was no cutoff number to define the diameter of arterioles and venules; their morphological appearance was assessed by comparison with reference images of the subpleural lung in patients with normal lung parenchyma; distal vessels were recognized as arterial or venous structures by sliding the CT sections above and below to identify their connection with recognizable arterial or venous divisions; they could also be recognized based on the location at the center/periphery of secondary pulmonary lobules when septal borders were depictable; (b) searched for the presence of CT features suggestive of microvasculopathy, comprising: (i) areas of focal abnormalities, categorized as ill-defined micronodules (i.e. small-sized, rounded [< 3 mm in diameter] areas of hyperattenuation, with blurred margins); lobular ground-glass opacities (GGO) (i.e., well-defined, rounded /triangular areas of GGO, identifying secondary pulmonary lobules filled by GGO on a transverse CT section); and haziness (i.e.*,* when areas of hyperattenuation did not fulfilled the morphology of the two previous categories); (ii) vascular tree-in-bud (i.e., dilated and irregular pulmonary arterioles); and (iii) neovascularity (i.e., dilated, serpiginous peripheral pulmonary vessels). Systemic-to-pulmonary anastomoses were analyzed as follows: (a) dilated peripheral systemic arteries, including intercostal arteries, internal mammary arteries and inferior phrenic arteries, were recognized in presence of small, serpiginous, vascular channels coursing in chest wall soft tissues in proximity to pleural surfaces; (b) the presence of systemic-to-pulmonary anastomoses was recognized when dilated peripheral systemic arteries were seen as “beading” along the visceral pleural margin and entering the lung to join intrapulmonary vessels, in the lung periphery.

### Conditions of image analysis

CT images were digitally stored and analyzed on a dedicated workstation (Syngo VIA, Siemens Healthineers). Two senior radiologists with 30 years of experience in chest CT and expertise in CTEPH selected the target areas and reviewed the images by consensus. The reading of images was preceded by training sessions to get familiar with the methodology and semiologic features to be analyzed. The readers were blinded to CTEPH characteristics (extent on CT images/hemodynamic data) and were not aware of the therapeutic options. According to the surgical classification of CTEPH, central disease extends from the pulmonary trunk to the proximal portion of segmental pulmonary arteries, while peripheral disease is characterized by clots only present within distal segmental and subsegmental arteries [22]. When chronic clots are exclusively located at the level of peripheral pulmonary arteries, the disease is then described as peripheral CTEPH [23].

### Statistical analysis

Statistical testing was conducted at the two-tailed α-level of 0.05. Data were analyzed using the SAS software version 9.4 (SAS Institute Inc). Categorical variables were expressed as frequencies and percentages. For quantitative variables, normality of distributions was assessed graphically and by using the Shapiro-Wilk test. As the normality assumption was accepted for all of them, the quantitative variables were expressed as mean ± standard deviation. For the primary objective, descriptive statistics were performed. For the secondary objectives, the comparisons between the two zones (hyper- and hypo- attenuating areas) were performed on the whole data set (i.e., all available pairs in the 29 patients) and according to available pairs of measures in several subgroups of patients (namely central and peripheral CTEPH and PAPm ≤ median value vs PAPm > median value). We used the general linear mixed model with patient as a random effect to take into account the correlation between observations within the same individual. We used binomial distribution and logit link except for pulmonary venules, where multinomial distribution was used.

## Results

### CT features of CTEPH

CT features of chronic PE were bilateral (*n* = 28; 96.6%) and unilateral (*n* = 1; 3.4%) findings, depicted at the level of central (mediastinal and/or lobar) and peripheral (segmental and/or subsegmental) pulmonary arteries (*n* = 22; 75.9%) and peripheral pulmonary arteries only (*n* = 7; 24.1%). Dilated bronchial and non-bronchial systemic arteries were seen in 26 patients (89.6%) and 18 patients (62.1%), respectively. On lung images (a) the gradient of attenuation between hypo- and hyper-attenuating areas of the mosaic perfusion pattern was rated as mild (*n* = 6; 20.7%) and marked (*n* = 23; 79.3%); (b) there were unilateral (*n* = 6; 20.7%) and bilateral (*n* = 3; 10.3%) features suggestive of sequelae of pulmonary infraction.

### CT findings in the entire study population (Table 2)

A total of 86 pairs were selected, including 3 pairs in 28 patients and 2 pairs in 1 patient. They were located in the right upper lobe (RUL) (*n* = 29), right middle lobe (*n* = 16), right lower lobe (*n* = 25), culmen (i.e., upper part of the left upper lobe) (*n* = 10), lingula (i.e., lower part of the left upper lobe) (*n* = 4) and left lower lobe (LLL) (*n* = 2).

**Table 2:** Comparison of CT findings at the level of 86 pairs in the study population of 29 patients

	Hyperattenuating areas *n* = 86	Hypoattenuating areas *n* = 86	p*
Morphology of anatomical structures			
Pulmonary arterioles			
Thin, *n* (%)	7 (8.1%)	**60** (**69.8%)**	**<** **0.001**
Dilated, *n* (%)	**79** (**91.9%)**	26 (30.23%)	
Pulmonary venules			
Thin, *n* (%)	15 (17.4%)	48 (55.8%)	**0.02**
Dilated, *n* (%)	**61** (**70.9%)**	31 (36.05%)	
Unremarkable, *n* (%)	10 (11.6%)	7 (8.1%)	
Septal lines, *n* (%)	14 (16.3%)	19 (22.1%)	0.51
CT abnormalities suggestive of microvasculopathy			
Focal lung infiltration			
Ill-defined micronodules, *n* (%)	19 (22.09%)	**35** (**40.70%**)	**0.008**
Lobular GGO, *n* (%)	2 (2.33%)	**13** (**15.1%)**	**0.01**
Haziness, *n* (%)	20 (23.3%)	**38** (**44.2%)**	**0.003**
Vascular tree-in-bud, *n* (%)	12 (14.0%)	17 (19.8%)	0.50
Neovascularity, *n* (%)	20 (23.3%)	13 (15.1%)	0.43
Subpleural lung			
Systemic-to-pulmonary anastomoses, *n* (%)	22 (25.6%)	31 (36.0%)	0.20

Bold characters reger to parameters with significant differences

NB: *p values for comparisons: the general linear mixed model with patient as a random effect was used to take into account the correlation between observations within the same individual. We used binomial distribution and logit link, except for pulmonary venules, where multinomial distribution was used

*GGO* ground-glass opacity

The diameter of peripheral pulmonary arteries significantly differed between the two zones (*p* < 0.0001), with dilated arterioles (*n* = 79/86; 91.9%) seen as the most frequent finding in hyperattenuating areas and thin arterioles (*n* = 60/86; 69.9%) in hypoattenuating areas.

**Fig. 2:**
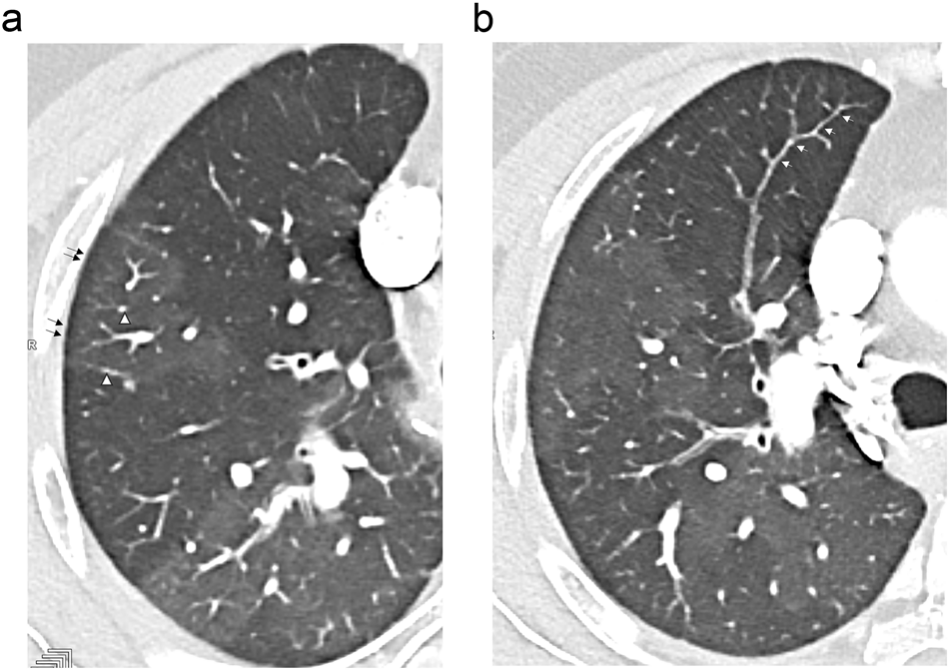
Two CT sections, spaced 10 mm apart in the right upper lobe (RUL), illustrating differences in diameter of distal pulmonary arterioles and venules between areas of hypo- and hyper-attenuation. **a** In the hyperattenuating area (arrows), note the presence of dilated centrilobular pulmonary arteries and depictable pulmonary veins in the interlobular septa (arrowheads). **b** In the adjacent hypoattenuated area, the presence of a thin pulmonary arterial section (arrows) extending to the subpleural zone

The distribution of pulmonary venule diameters significantly differed (*p* = 0.02) between the two zones, with a majority of dilated venules in hyperattenuating areas (*n* = 61/86; 70.9%) and a majority of thin venules in hypoattenuating areas (*n* = 48/86; 55.8%) (Fig. 2).

There was no significant difference in the frequency of detectable septal lines between hyper- and hypo-attenuated zones (*p* = 0.51).

The frequency of ill-defined micronodules (*n* = 35; 40.70% vs n = 19; 22.09%; *p* = 0.008), lobular GGO (*n* = 13; 15.12% vs *n* = 2; 2.33%; *p* = 0.01) and haziness (*n* = 38; 44.2%; *p* = 0.003) was significantly higher in hypoattenuated areas while there was no significant difference in the frequency of neovascularity between the two area (*p* = 0.43).

**Fig. 3:**
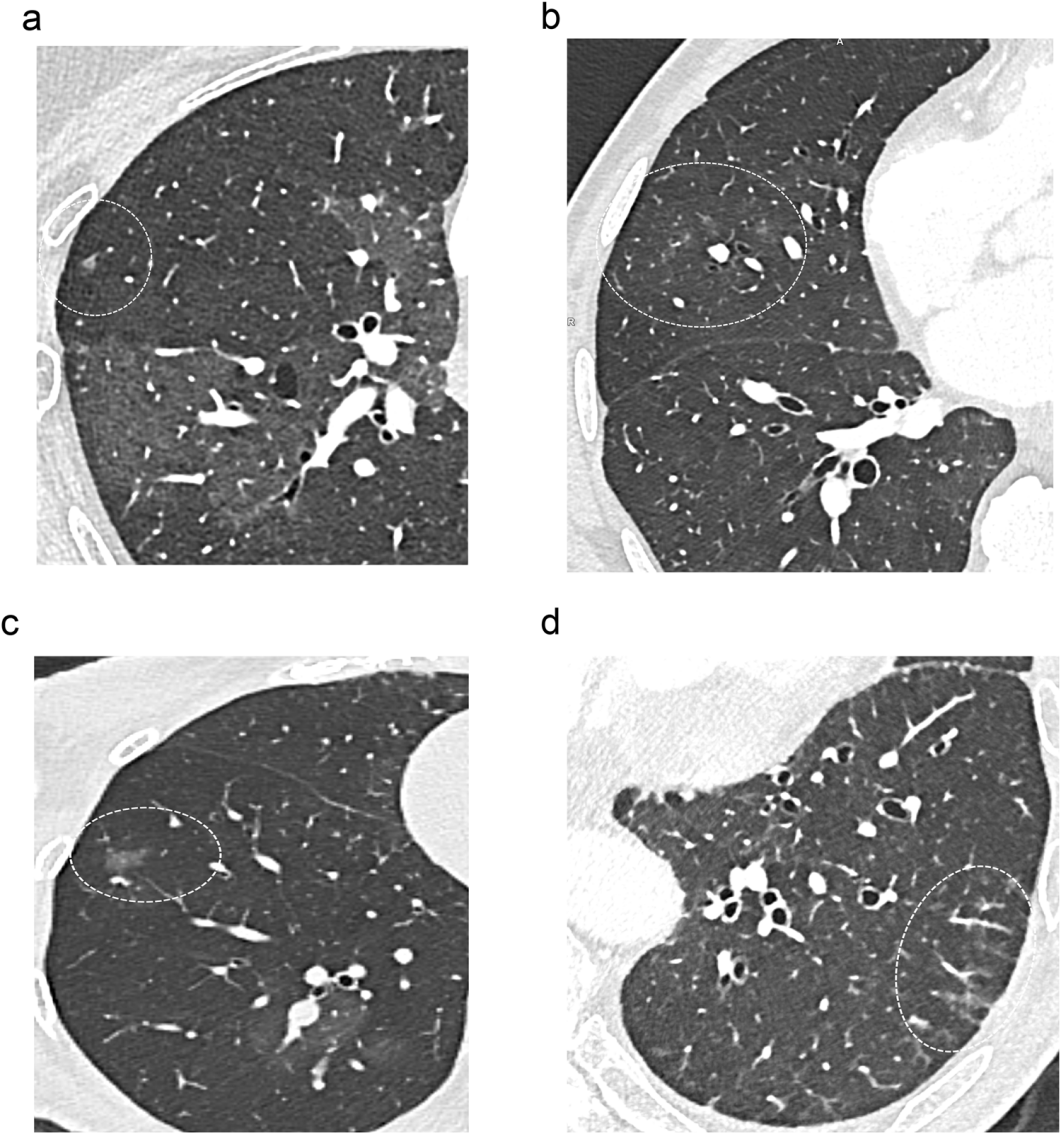
Transverse CT sections from different patients illustrating the different aspects of areas of increased attenuation (dotted circles), seen as focal (**a**) or numerous ill-defined (**b**) micronodules, lobular ground-glass attenuation (**c**), and haziness (**d**)

**Fig. 4:**
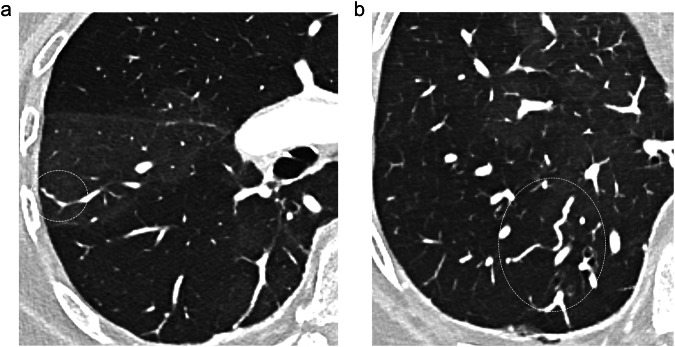
Transverse CT sections from two patients illustrating the appearance of neovascularity, seen as a dilated and beaded subpleural vessel in **a** (dotted circle) and a tortuous, enlarged vessel in **b** (dotted circle)

**Fig. 5:**
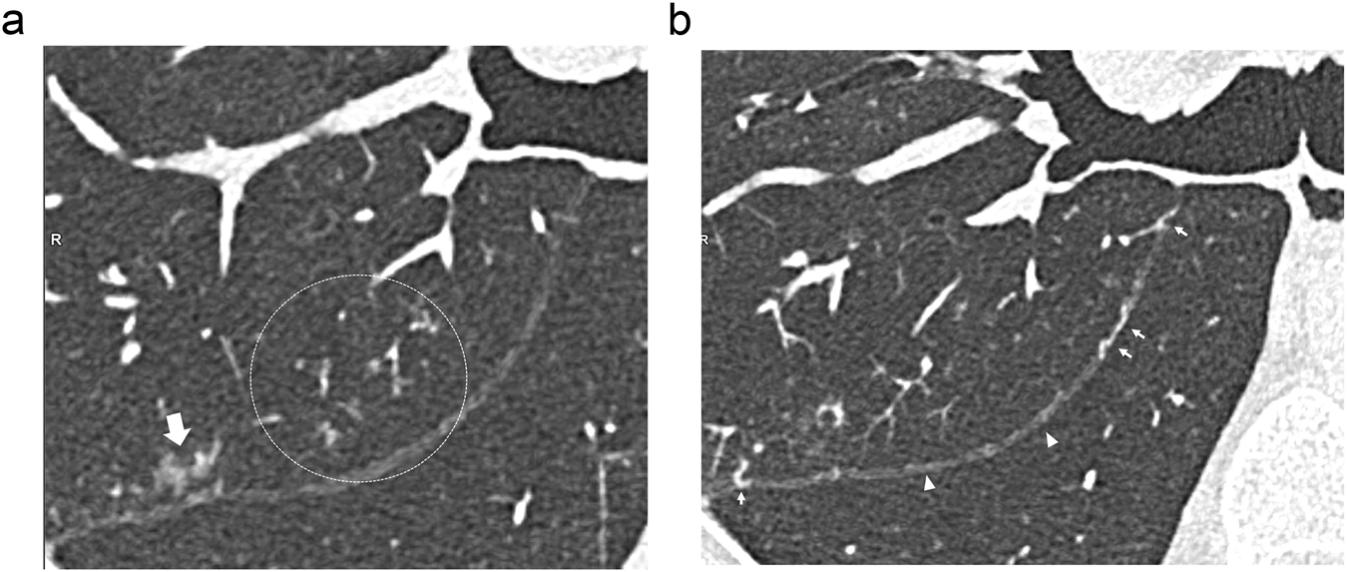
Two CT sections from the same patient illustrating a vascular tree-in-bud in (**a**) (dotted circle), seen in the vicinity of dense, ill-defined micronodules (large arrow) in the posterior segment of the RUL. On the transverse CT section obtained 3 mm below (**b**), note the presence of dilated tortuous pulmonary vessels (arrows) joining and coursing within the upper part of the right major fissure, otherwise thickened (arrowheads)

**Fig. 6:**
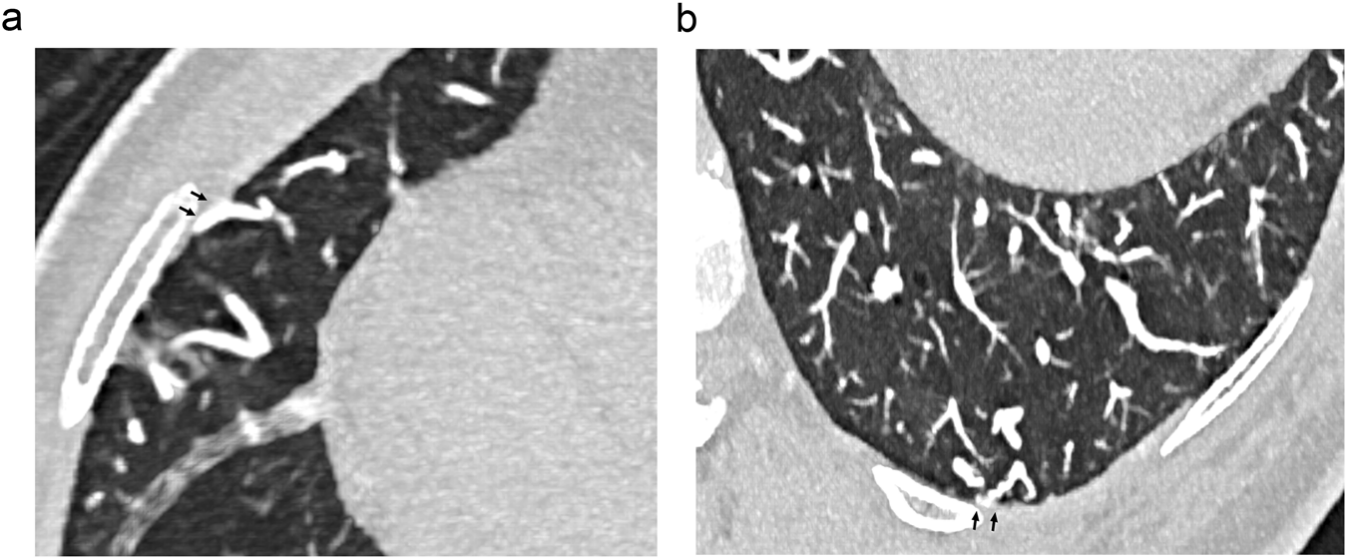
Three-mm thick maximum intensity projections from the same patient scanned with UHR spectral CTA, illustrating the presence of numerous systemic-to-pulmonary anastomoses in the right middle lobe (**a**) and LLL (**b**). Communications are seen between dilated peripheral intercostal arteries and enlarged pulmonary vascular sections in the subpleural areas (arrows)

The frequency of vascular tree-in-bud (*p* = 0.50) and systemic-to-pulmonary anastomoses (*p* = 0.20) did not differ between hypo- and hyper-attenuating areas. A panel of illustrations (Figs. 3–6) summarizes the CT features identified in the subpleural lung.

### CT findings in the population stratified by the extent of chronic clots (Table 3)

In the 22 patients with CT features of central and peripheral CTEPH, we compared CT findings at the level of 66 pairs and observed (Table 3A): (a) similar significant differences in the diameters of arterioles and venules between areas of hypo- and hyper-attenuation as those described in the entire study population; (b) in hypoattenuated areas, there was a higher frequency of lobular GGO (*p* = 0.01) and haziness (*p* = 0.04) the frequency of vascular tree in bud (*p* = 0.09), neovascularity (*p* = 0.50) and systemic-to-pulmonary anastomoses (*p* = 0.43) did not differ between the two zones.

**Table 3:** (A) Comparison of CT findings at the level of 66 pairs in 22 patients with central and peripheral CTEPH. (B) Comparison of CT findings at the level of 20 pairs in 7 patients with peripheral CTEPH

A			
	Hyperattenuating areas *n* = 66	Hypoattenuating areas *n* = 66	p*
Morphology of anatomical structures			
Pulmonary arterioles			
Thin, *n* (%)	5 (7.6%)	**43 (65%)**	**<** **0.001**
Dilated, *n* (%)	**61 (92.4%)**	23 (34.8%)	
Pulmonary venules			
Thin, *n* (%)	14 (21.2%)	**35 (53%)**	**0.001**
Dilated, *n* (%)	**47 (71.2%)**	27 (40.9%)	
Unremarkable, *n* (%)	5 (7.6%)	4 (6.1%)	
Septal lines, *n* (%)	11 (16.7%)	15 (22.7%)	0.63
CT abnormalities suggestive of microvasculopathy			
Focal lung infiltration			
Ill-defined micronodules, *n* (%)	19 (22.09%)	29 (44%)	0.08
Lobular GGO, *n* (%)	2 (2.33%)	**13 (19.7%)**	0.01
Haziness, *n* (%)	20 (23.3%)	**32 (48.5%)**	0.04
Vascular tree-in-bud, *n* (%)	9 (13.6%)	16 (24.2%)	0.09
Neovascularity, *n* (%)	17 (25.8%)	12 (18.2%)	0.50
Subpleural lung			
Systemic-to-pulmonary anastomoses, *n* (%)	19 (28.8%)	26 (39.4%)	0.43

B			
	Hyperattenuating areas *n* = 20	Hypoattenuating areas *n* = 20	p*
Morphology of anatomical structures			
Pulmonary arterioles			
Thin, *n* (%)	2 (10%)	**17 (85%)**	**<** **0.001**
Dilated, *n* (%)	**18 (90%)**	3 (15%)	
Pulmonary venules			
Thin, *n* (%)	1 **(**5%)	**13 (65%)**	**0.049**
Dilated, *n* (%)	**14 (70%)**	4 (20%)	
Unremarkable, *n* (%)	5 (25%)	3 (15%)	
Septal lines, *n* (%)	3 (15%)	4 (20%)	0.62
CT abnormalities suggestive of microvasculopathy			
Focal lung infiltration			
Ill-defined micronodules, *n* (%)	0	6 (30%)	NC^+^
Lobular GGO, *n* (%)	0	0	NA
Haziness, *n* (%)	0	6 (30%)	NC^+^
Vascular tree-in-bud, *n* (%)	3 (15%)	1 (5%)	0.07
Neovascularity, *n* (%)	3 (15%)	1 (5%)	0.59
Subpleural lung			
Systemic-to-pulmonary anastomoses, *n* (%)	3 (15%)	5 (25%)	0.20

Bold characters reger to parameters with significant differences

NB: *p values for comparisons: the general linear mixed model (GLMM) with patient as a random effect was used to take into account the correlation between observations within the same individual. We used binomial distribution and logit link except for pulmonary venules, where multinomial distribution was used

NC^+^: the GLMM model does not converge due to too small samples

*GGO* ground-glass opacity

In the seven patients with peripheral CTEPH, the comparison of 20 pairs showed (Table 3B): (a) similar differences in the diameters of arterioles and venules between areas of hypo- and hyper-attenuation to those described in the entire study population; (b) the absence of ill-defined micronodules, lobular GGO and haziness in hyperattenuated areas; and (c) the absence of lobular GGO in hypoattenuated areas. The frequency of vascular tree-in-bud (*p* = 0.07) and systemic-to-pulmonary anastomoses (*p* = 0.59) did not differ between the two zones.

### CT findings in the population stratified by PAPm (Table 4)

The population was divided into two subgroups of patients, stratified by the median value of mean pulmonary artery pressure (PAPm) in the entire study group (i.e., 42 mm Hg).

**Table 4:** (A) Comparison of CT findings at the level of 45 pairs in 15 patients with a mean PAP ≤ 42 mm Hg. (B) Comparison of CT findings at the level of 41 pairs in 14 patients with a mean PAP > 42 mm Hg

A			
	Hyperattenuating areas *n* = 20	Hypoattenuating areas *n* = 20	p*
Morphology of anatomical structures			
Pulmonary arterioles			
Thin, *n* (%)	1 (2.2%)	**34** **(75.6%)**	**<** **0.001**
Dilated, *n* (%)	**44** **(97.8%)**	11 (24.4%)	
Pulmonary venules			
Thin, *n* (%)	7 (15.6%)	24 (53.3%)	0.08
Dilated, *n* (%)	35 (77.8%)	14 (31.1%)	
Unremarkable, *n* (%)	3 (6.7%)	7 (15.6%)	
Septal lines, n (%)	7 (15.6%)	9 (20.0%)	0.51
CT abnormalities suggestive of microvasculopathy			
Focal lung infiltration			
Ill-defined micronodules, *n* (%)	8 (17.8%)	**22** **(48.9%)**	**0.003**
Lobular GGO, *n* (%)	1 (2.2%)	8 (17.8%)	NC^+^
Haziness, *n* (%)	8 (17.8%)	**25** **(55.6%)**	**<** **0.001**
Vascular tree-in-bud, *n* (%)	4 (8.9%)	2 (4.4%)	0.69
Neovascularity, *n* (%)	10 (22.2%)	7 (15.6%)	0.76
Subpleural lung			
Systemic-to-pulmonary anastomoses, *n* (%)	11 (24.4%)	**21 (46.7%)**	**0.02**

B			
	Hyperattenuating areas *n* = 41	Hypoattenuating areas *n* = 41	
Morphology of anatomical structures			
Pulmonary arterioles			
Thin, *n* (%)	6 (14.7%)	**26** **(63.4%)**	**<** **0.001**
Dilated, *n* (%)	**35** **(85.4%)**	15 (36.6%)	
Pulmonary venules			
Thin, *n* (%)	8 (19.5%)	**24** **(58.5%)**	**0.001**
Dilated, *n* (%)	**26** **(63.4%)**	17 (41.5%)	
Unremarkable, *n* (%)	7 (17.1%)	0	
Septal lines, *n* (%)	7 (17.1%)	10 (24.4%)	0.74
CT abnormalities suggestive of microvasculopathy			
Focal lung infiltration			
Ill-defined micronodules, *n* (%)	11 (26.8%)	13 (31.8%)	0.56
Lobular GGO, *n* (%)	1 (2.4%)	5 (12.2%)	0.12
Haziness, *n* (%)	12 (29.3%)	13 (31.7%)	0.75
Vascular tree-in-bud, *n* (%)	10 (24.4%)	13 (31.7%)	0.57
Neovascularity, *n* (%)	10 (24.4%)	6 (14.7%)	0.26
Subpleural lung			
Systemic-to-pulmonary anastomoses, *n* (%)	11 (26.8%)	10 (24.3%)	0.46

Bold characters reger to parameters with significant differences

NB: *p values for comparisons: the general linear mixed model with patient as a random effect was used to take into account the correlation between observations within the same individual. We used binomial distribution and logit link except for pulmonary venules, where multinomial distribution was used

NC^+^: the GLMM model does not converge due to too small samples

*GGO* ground-glass opacity

There were 15 patients with a PAPm ≤ 42 mm Hg (mean PAPm: 35.9 ± 5.1 mm Hg) in whom we compared CT findings at the level of 45 pairs (Table 4A): (a) similar significant differences in the diameters of arterioles between areas of hypo- and hyper-attenuation as those described in the entire study population (*p* < 0.001), while no significant difference was found for the frequency of venules (*p* = 0.08); (b) in hypoattenuated areas, there was a significantly higher frequency of ill-defined micronodules (*p* = 0.003) and haziness (*p* < 0.001), as well as a higher frequency of systemic-to-pulmonary anastomoses (*p* = 0.02); (c) there was no difference in the frequency of vascular tree-in-bud (*p* = 0.69) and neovascularity (*p* = 0.76). The Frequency of lobular GGO could not be compared due to too small sample size.

There were 14 patients with a PAPm > 42 mm Hg (mean PAPm: 52.8 ± 5.7 mm Hg) in whom we compared the CT findings at the level of 41 pairs. As shown in Table 4B, we observed (a) similar significant differences in the diameters of arterioles and venules between areas of hypo- and hyper-attenuation to those described in the entire study population; (b) the absence of significant differences in the frequency of all tested CT features.

## Discussion

To our knowledge, this is the first study reporting morphologic features at the level of the subpleural lung of CTEPH patients examined with high-resolution CT at 0.2 mm and 0.4 mm collimation. Comparing the findings in 86 pairs of lung parenchyma with mosaic perfusion, we observed significant differences in the diameters of the most distal pulmonary vessels with a high frequency of dilated arterioles (91.9%) and venules (70.9%) in areas of GGO, while thin arterioles and venules were the most frequent findings in areas of hypoattenuation, respectively depicted in 69.8% and 55.8% of the studied areas. These findings add morphological evidence to the well-known differences in pulmonary perfusion within each area of mosaic perfusion in CTEPH.

In the overall study population, the first interesting finding was to observe that the CT features extrapolated from pathophysiologic descriptions of CTEPH microvasculopathy could be visually detectable in the subpleural lung. We depicted ill-defined micronodules, similar to those described in idiopathic and hereditary PAH [24–27], and also seen in PVOD/PCH [28, 29]. Larger areas of GGO, rated as lobular GGO in the present study, were also identified. From a semantic standpoint, our findings can be compared to the lobular ground-glass pattern reported in the early descriptions of CT findings in Eisenmenger syndrome [30] and ground-glass halos adjacent to pulmonary vessels recently reported in PAH [27, 30, 31], while lobular GGO is also described in PVOD/PCH [28, 29, 32]. More difficult was the characterization of areas of increased attenuation that did not strictly follow standard descriptions of ill-defined micronodules and lobular GGO. They were seen as larger areas of increased attenuation through which we identified vessels often seen with blurred margins; we gathered such features under the term haziness. These three CT features were seen with a significantly higher frequency in hypoattenuated areas. Whereas this might be related to an easier detectability in the surrounding low-attenuation background, one could also link their higher frequency to the presence of more severe microvasculopathy in areas of hypoperfusion.

We observed tortuous and dilated subpleural vessels, originally described as neovascularity in patients with Eisenmenger syndrome [30] but also in patients with idiopathic pulmonary arterial hypertension [33]. Whereas their frequency did not differ between hypo- and hyper-attenuating areas, their detectability in CTEPH suggests another analogy with PAH-like lesions. Such peripheral neovascularity could also be considered as an equivalent of the increased tortuosity that can be demonstrated at the level of more central vessels when using 3D reconstructions of the pulmonary vasculature [34]. An intriguing finding was observed at the level of distal arterioles. Beyond a severely stenosed arteriolar portion, the arteriole was seen with a beaded and somewhat dilated appearance that we described as vascular tree-in-bud. Based on pathophysiologic descriptions, we hypothesize that this might reflect the recanalization of occluded arteries by dilated bronchial arteries, known to occur via vasa-vasorum [35].

Regarding systemic-to-pulmonary anastomoses, we observed two areas of abnormal communications. In the perithoracic subpleural lung, we could identify enlarged non-bronchial systemic arteries (i.e., intercostal, internal thoracic, and inferior phrenic arteries) entering the subpleural lung with connections to pulmonary vessels. Similar subpleural connections were also seen between pulmonary vessels and dilated intra-fissural vessels, very likely to correspond to dilated bronchial arteries. Whereas we did not find significant differences in the frequency of systemic-to-pulmonary anastomoses between hypo-and hyper-attenuating areas in the entire study group, they were depicted more frequently in the periphery of hypoattenuated areas (36% vs 25.6%). At a patient level, a recent study showed that the distribution of systemic-to-pulmonary collaterals was positively correlated with perfusion defects in the lung segments of CTEPH [36]. These authors also underlined the role of these collaterals, known to primarily perfuse into distal occluded lung areas, and representing major contributors to small-vessel disease in this area [35].

Additional analyses were then undertaken to determine whether we could nuance CT findings by CTEPH characteristics. In all subgroup analyses, we observed that the main characteristics regarding the diameters of pulmonary arterioles and venules in hypo- and hyper-attenuating areas described in the entire study population were present. In patients with central and peripheral CTEPH, we observed that lobular GGO and haziness were significantly more frequent in hypoattenuated areas, whereas ill-defined micronodules did not differ between the two areas. In patients with peripheral CTEPH, the intriguing finding was the absence of ill-defined micronodules, lobular GGO, and haziness in hyperattenuating areas, raising limitations in the depiction of subtle changes on CT images. When stratifying the population by PAPm, the subgroup of patients with a PAPm ≤ 42 mm Hg showed a higher frequency of ill-defined micronodules and haziness in hypoattenuating areas. In addition, a significantly higher frequency of systemic-to-pulmonary anastomoses was also found in these areas, suggesting an important development of systemic collaterals in this hemodynamic subgroup. Regarding patients with PAPm > 42 mm Hg, all CT features suggestive of microvasculopathy did not differ between hypo- and hyperattenuating areas, suggesting homogenization of CT findings in the mosaic perfusion pattern.

Several limitations of this study deserve mention. First, this study was retrospective and performed at a single center in a small group of patients. This resulted from an early experience with PCD-CT in clinical routine. Second, there was no unique scanning protocol for lung analysis, which obtained 0.2 mm and 0.4 mm-thick images, the former fulfilling the criteria of «ultra-high-resolution». However, one should underline that the spatial resolution of lung images in all PCD-CT examinations was superior to that available with energy-integrating-detector CT scanners. Third, the number of subpleural zones considered for our analysis did not exceed three pairs per patient. Whereas we could have selected a greater number of targets, we considered that their selection not only in different anatomical zones but also in lung parenchymal areas fully analyzable could represent a reliable sample for this preliminary study. Fourth, the existence of microvasculopathy was not verified by histological examination, and our findings simply reflect a semiology deemed to be reasonably detectable on PCD-CT lung images. Further studies based on pathologic-CT correlations are mandatory to confirm present data. Fifth, the reading of images was obtained by consensus, which did not allow for calculating inter- and intra-observer agreement. Because this study was designed as a preliminary experience aimed at detecting subtle CT features using a newly introduced CT technology, we favored the consensus between two radiologists with a longstanding knowledge of PH-related changes and experience in PCD-CT. Lastly, two methodological limitations must be underlined.

The analysis of pairs could not be blinded, raising a limitation inherent to the visual evaluation with a risk of recall bias. However, one should underline that there was quite a long list of subtle CT features to analyze in each target area. This imposed on the readers meticulous, time-consuming readings with no attempt to add comparative analysis of findings from lobe to lobe. One could also raise concerns about potential bias in the selection of pairs. However, their selection was exclusively dictated by their analyzability on transverse CT sections, requiring clear depiction of the mosaic perfusion pattern and lack of artifacts, either due to cardiac/respiratory motion or sequelae of pulmonary infarction. Lastly, because no correction for multiple testing was done, we cannot exclude false-positive results. However, one should mention that the present study is an exploratory study; results should be subsequently interpreted with caution and considered as hypothesis-generating.

In conclusion, our study demonstrates the detectability of subtle morphological changes in the subpleural lung, extrapolated from pathophysiological descriptions. These preliminary results raise high expectations from PCD-CT in the non-invasive approach of microvascular changes in CTEPH.

## Supplementary information

ELECTRONIC SUPPLEMENTARY MATERIAL

## Abbreviations

BPA: Balloon pulmonary angioplasty
CTEPH: Chronic thromboembolic pulmonary hypertension
GGO: Ground-glass opacities
LLL: Left lower lobe
PAH: Pulmonary artery hypertension
PAPm: Mean pulmonary artery pressure
PEA: Pulmonary thromboendarterectomy
RUL: Right upper lobe
