# Supplementary material for: Lung microvasculopathy in chronic thromboembolic pulmonary hypertension: high-resolution findings with photon-counting detector CT in 29 patients
Source: Eur Radiol. 2025 Apr 18;35(10):6369–81. doi: 10.1007/s00330-025-11561-w (PMC12417283; doi:10.1007/s00330-025-11561-w)
Supplement: Supplementary file 1 — ELECTRONIC SUPPLEMENTARY MATERIAL [file 330_2025_11561_MOESM1_ESM.pdf]

Lung microvasculopathy in chronic thromboembolic pulmonary hypertension: high-resolution findings with photon-counting detector CT in 29 patients

ELECTRONIC SUPPLEMENTARY MATERIAL

Table A: Scanning parameters

**Acquisition parameters**	**UHR**<br>**non-contrast**<br>n=9	**UHR**<br>**Spectral CTA**<br>n=11	**Standard**<br>**Spectral CTA**<br>n=9
X-ray source	Single-source	Single-source	Single-source<br>Dual-source
Kilovoltage	Sn100 / Sn 140 depending on patient morphotype	140	120 / 140
Collimation	120 x 0.2 mm	120 x 0.2 mm	144 x 0.4 mm
Pitch	1	1	1.5/ 2-3
Rotation time*, s*	0.25	0.25	0.25
IQ level	36<br>*(ref mAs : 66)*	80<br>*(ref mAs : 57)*	80<br>*(ref mAs : 57)*
CAREDose 4D	on	on	on
Caudo-cranial acquisitions	+	+	+
End-inspiratory acquisition	+	+	+
**Injection parameters**			
Iodine concentration, *mg I/mL*		400	400
Flow rate*, mL/s*		4	4
Volume administered, *mL*		80 *(iodine)* + 40 *(diluted contrast medium)*	80 *(iodine)* + 40 *(diluted contrast medium)*
ROI position		ascending aorta	ascending aorta
Threshold, *HU*		150<br>*(on serial acquisitions at 90 kVp)*	150<br>*(on serial acquisitions at 90 kVp)*
**Reconstruction parameters for lung images**			
-thickness, *mm*	0.2	0.2	0.4

-intervals, *mm*<br><br>-kernel<br><br>-level of iterative reconstruction	0.2<br><br>Bl60<br><br>QIR 4	0.2<br><br>Bl60<br><br>QIR 3	0.4<br><br>Bl60<br><br>QIR 3
Energy level for monoenergetic reconstruction	T3D	T3D	70 keV

Abbreviations: UHR: ultra-high-resolution; CTA: computed tomographic angiography; IQ: ; 4D: four dimensional; HU: Hounsfield Unit; keV: kilo-electron-volt; QIR: quantum iterative reconstruction

Supplementary Material -

Table B

From pathophysiology descriptions to CT features of microvasculopathy in CTEPH

	**Pathophysiological findings**	**Observed pathological lesions**		**Extrapolated CT features**
**Areas of nonobstructed pulmonary arteries** *(hyperattenuating areas on CT)*	-redistribution of blood flow<br>-PA exposed to high pressure and shear stress	PAH-like lesions including intimal thickening and remodelling of pulmonary resistance vessels, eccentric intimal fibrosis, intimal fibromuscular proliferation and plexiform lesions.	→	-dilated arterioles<br>-subpleural tortuosity ***(*****25,28,29,31*****)***<br>-ill-defined micronodules (**22-25**);
**Areas of obstructed pulmonary arteries** *(hypoattenuating areas on CT)*	*Distally to pulmonary arteries occluded by fibrotic material:*<br><br>(a) recanalization by bronchial arteries	(a) recanalization via vasa vasorum	→<br><br>→	-PA recanalized: *dilated and irregular pulmonary arterioles (described as vascular tree-in-bud)*<br><br>-PA not recanalized: *arterioles of small diameter*
	(b) anastomoses between bronchial arteries and the pulmonary circulation	(b) anastomosis at the:<br>-precapillary level, responsible for PAH-like lesions;<br><br>-capillary & and venular levels: PVOD/PCH lesions	→<br><br>→	PAH-like lesions *(cf above)*<br><br>lobular GGO & septal lines (**26,27)**
	(c) creation of subpleural systemic-pulmonary anastomoses.		→	Signs suggestive of systemic-to-pulmonary anastomoses in the subpleural region.
